# Review: Acute superior mesenteric artery embolism: A vascular emergency cannot be ignored by physicians

**DOI:** 10.1097/MD.0000000000014446

**Published:** 2019-02-08

**Authors:** Guanyi Liao, Siyang Chen, Haoyang Cao, Wuwan Wang, Qing Gao

**Affiliations:** a Department of Gastroenterology; b Department of Cardiology, the First Affiliated Hospital, Chongqing Medical University, Chongqing, China.

**Keywords:** acute mesenteric ischemia, diagnostic approach, therapeutic management

## Abstract

**Background:**

Acute mesenteric ischemia (AMI) is a life-threatening medical condition that occurs when a sudden decreased perfusion to the intestines which leads to bowel infarction, and acute superior mesenteric artery embolism (ASMAE) is the main cause of AMI. Unfortunately, with the improvement of diagnosis and treatment technology, the mortality remains high due to less frequent clinical suspicion resulted from the unclear clinical manifestation and non-specific laboratory findings.

**Methods:**

Relevant studies published were identified by searching the PubMed, Embase and Cochrane Library databases. This review presented the literatures to introduce the research progress of ASMAE in recent years.

**Results:**

Patients with the history of atrial fibrillation, heart valve disease and atherosclerosis should be considered as ASMAE. Laboratory findings are insensitive and unspecific, however, angiography and Computed tomography angiography (CTA) can provide a clear diagnosis sensitively and specifically. Endovascular approaches have been increasingly reported in multiple case series. The key to successful treatment of AMI involves early clinical recognition and early intervention to move the embolus, which can reduce the rate of misdiagnosis and save the precious time and lives of patients.

**Conclusions:**

Loss of time eventually leads to progression of ischemia to transmural bowel necrosis with peritonitis and septicemia, which may further worsen patients’ outcomes. It is important for physicians to make a timely and accurate diagnosis, which can save precious time and reduce the mortality.

## 1 Introduction

Acute mesenteric ischemia (AMI), which is defined as a sudden decreased perfusion to the intestines which commonly results in bowel infarction, is an uncommon medical condition with high mortality rate from 50% to 70%, accounting for 1% to 2% of acute abdominal emergencies. AMI includes inadequate blood supply, inflammatory injury and finally necrosis of the bowel wall, and can occur as a result of arterial embolism, arterial thrombosis, mesenteric venous thrombosis and non-occlusive causes. Acute superior mesenteric artery embolism (ASMAE), the main subtype of AMI, accounts for 40% to 50% of the AMI cases.^[1–2]^ ASMAE generally presents as severe and acute-onset abdominal pain with the lack of obvious and specific signs, and is usually diagnosed at an advanced stage.

Mesenteric angiography was the gold standard in the past for the diagnosis of ASMAE, which was recommended by The American Gastroenterological Association.^[3]^ However, angiography is time consuming and invasive, with unavailability in many hospitals, which can lead to critical delay. As a result, angiography is superseded by CT angiography in diagnosing ASMAE, as it is noninvasive and 24hour-accessible, with a high sensitivity and specificity of 0.96 and 0.94, respectively.^[4]^ Once the diagnosis has been confirmed, surgical operation is the most popular choice if contraindications have been excluded. Alleviation of intestinal ischemia and resection of necrotic bowel is the principle of laparotomy operation.^[5]^ Moreover, with the rapid progress in therapeutic efficacy of interventional treatment, it tends to be a better choice for helping patients to recover in a favoring way, with little invasion and less time.

However, ASMAE still has a high mortality rate because of being usually diagnosed at an advanced stage. In clinical practice, patients with ASMAE are not frequently seen and has no specific symptoms and signs at the initial stage, or the clinician does not have sufficient understanding on differential diagnosis of abdominal pain and miss out on diagnosis of ASMAE timely. By the time diagnosis of ASMAE is made, the disease progresses to irreversible intestinal obstruction and gangrene, resulting in the increase of mortality despite the major diagnostic and treatment advances over the past decades. As a result, early recognition and prompt treatment are crucial for survival and prognosis.^[6]^ Although either laparotomy or interventional operation of ASMAE is frequently accomplished by vascular surgeons, it is still essential to enhance physicians’ knowledge and strengthen physicians’ awareness about this disease so that these patients may be admitted into the department of gastroenterology, which perhaps have the most rapid clinical decline without timely accurate diagnosis.

## 2 Etiology and risk factor

Superior mesenteric artery (SMA) is grafted from abdominal aorta with an acute angle, and has a great diameter so that the embolus can easily flow into the SMA with the blood flow.^[4]^ The embolus lodging in the SMA is mainly from a cardiac source, which is common in patients with valvular heart disease, coronary heart disease, bacterial endocarditis, prior myocardial infarction, and atrial fibrillation.^[6]^ In some unusual cases, the detached atherosclerotic plaque, mural thrombus in the aneurysm and venous thrombosis can also cause ASMAE.^[4]^ Furthermore, Amol et al^[7]^ have even reported a case of SMA embolism resulted from Q fever endocarditis of the aortic valve.

The most important risk factors for ASMAE are the time to diagnosis and restoration of the blood supply, the location of embolus, patient age and comorbidities. The outcome of ASMAE is closely dependent on the elapsed time to diagnosis and treatment. Ha et al^[8]^ think that the substantial time for ischemic bowel is 12 hours, without any permanent injuries. In most cases of ASMAE, emboli lodge about 6 to 8 cm beyond the SMA origin, distal to the origin of the middle colic artery. Nevertheless, atheroembolic emboli are more likely to be smaller so they could lodge in the more distal mesenteric circulation, which perhaps has a better prognosis. Patients are often present in their 60 to 70 seconds and are easily associated with a variety of medical comorbidities. It has been reported by Yusuf et al^[9]^ the patients’ mean age in their retrospective study was 68.43 years. Recently, a prospective study conducted by Nuzzo et al^[10]^ identified three predictive factors for irreversible transmural intestinal necrosis (ITIN) in AMI, including organ failure, serum lactate levels and bowel loop dilation on computerized tomography scan. In this study, ITIN rate increased from 3% to 38%, 89%, and 100% in patients with 0, 1, 2, and 3 factors, respectively.

## 3 Pathophysiology

SMA, which is grafted from abdominal aorta with an acute angle, supplies the blood to the entire small intestine, ascending colon and part of the transverse colon. The SMA has a great diameter and is parallel with abdominal aorta so that the embolus can easily pass through the SMA with the blood flow. The distal blood supply will be interrupted partially or completely pertaining to the diameter of embolism in the different branches of blood vessels or bifurcation, which brings about intestinal ischemia, edema, necrosis, and perforation.^[11]^

**Figure 1:**
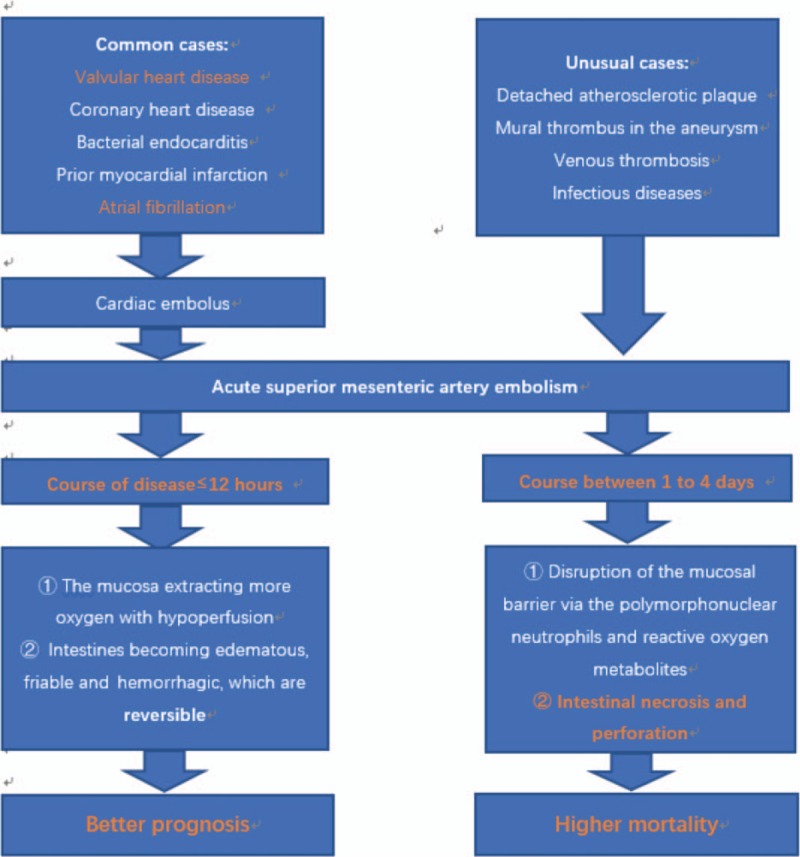
The pathophysiology of acute superior mesenteric artery embolism.

The mucosal surfaces are the first to be affected by the high metabolic demand compared to the serosa. During initial stages, the walls of involved intestines undergo congestion and then it becomes edematous, friable, and hemorrhagic. Proposed mechanisms lead to the preservation of splanchnic tissue perfusion, direct arteriolar smooth muscle relaxation and a metabolic reaction of adenosine and other metabolites of mucosal ischemia. Moreover, in order to preserve the integrity of mucosa during periods of metabolic insult, the intestinal mucosa can extract increasing amounts of oxygen in the condition of hypoperfusion. And persistent ischemia results in disruption of the mucosal barrier via the action of polymorphonuclear neutrophils and reactive oxygen metabolites.^[12]^ Without emergency treatment, patients possibly would present with bowel hemorrhage within 1 to 4 days because of enteric bacteria resulting from mucosal barrier collapse leading to gangrene which causes perforation and sometimes severe sepsis or multiorgan failure^[11,13]^ (Fig. 1).

## 4 Clinical manifestation

Clinically, ASMAE is characterized by an abrupt onset of severe abdominal pain disproportionate to the physical examination findings because there is transmural involvement of the bowel when there is relatively little peritoneal irritation. The abdominal pain is the primary symptom and initially described as constant, diffuse, non-localized or periumbilical, cramp-like abdominal pain, usually difficult to be relieved by antispasmodic agents.^[11]^ Moreover, ASMAE has the most rapid clinical decline due to the lack of established collateral circulation. It is pivotal to note that abdominal pain is usually followed by a deceptive pain-free interval because of a decline in intramural pain receptors owing to sustained under-perfusion of the intestinal wall.^[14]^ Peritonitis and septicemia develop once the ischemia and necrosis have progressed transmurally.

Other symptoms are present inconstantly and include vomiting, diarrhea, nausea, abdominal distension, fever, and rectal bleeding. In the early stage, the reduction of blood flow can lead to intestinal edema and weakened peristalsis resulting in vomiting and diarrhea, which is called gastric emptying disorder. It has been proved that vomiting (71%) and diarrhea (42%) are correlated in contemporary prospective study.^[15]^ Generally, the vomitus is gastric contents because the stomach blood supply is not provided by SMA. If patients present with hematemesis, other causes of hematemesis such as stress ulcer and medicine (NSAIDS) should be considered.

Examination findings early in the course of the disease are limited and non-specific. Hyperactive bowel sounds without apparent tenderness, rebound tenderness and guarding may be found within 12 hours after the onset. Later, the bowel sounds weaken or disappear and peritoneal irritation sign begins to be obvious, which are induced by many metabolic products due to the irreversible intestinal necrosis (Table 1).

**Table 1:**
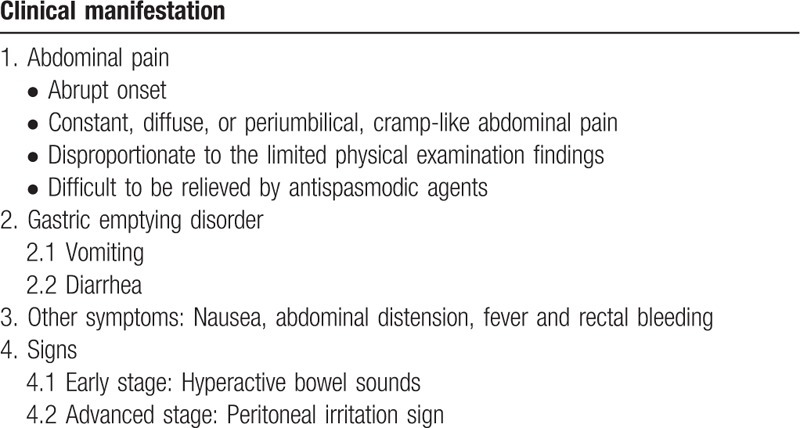
The clinical manifestation of acute superior mesenteric artery embolism.

Abdominal pain is easy to be mistaken for other more common diseases, such as pancreatitis, appendicitis, cholecystitis, bowel obstruction, and diverticulitis. Mismatch of severe symptoms and unobvious physical examinations, history of organic heart disease or atrial fibrillation and manifestation of gastric emptying disorder is known as Bergan triad.^[16]^ However, physicians should keep in mind that not every patient presents with the Bergan triad.

## 5 Laboratory findings

Classically, patients with ASMAE have leukocytosis, metabolic acidosis, an elevated D-dimer and serum lactate. Elevated level of C-reactive protein (CRP), creatine kinase, hemoconcentration and a high anion gap in severe cases are very common. In some cases, high amylase, aspartate aminotransferase and dehydrogenase can also be found in laboratory findings.^[12,17]^ A readily accessible, simple, highly sensitive and specific serum maker to identify patients with ASMAE timely would be of great significance in choosing candidates for Computed tomography angiography (CTA).

Recently, due to elevations in some AMI serum makers often present after extensive transmural bowel infarction, such as L-lactate, an ideal biomarker for ASMAE ought to originate at the mucosa to detect ischemia at the earliest stage because ischemia begins at the mucosa and extends toward the serosa.^[18]^ However, a relationship between serum lactate and the extent of bowel ischemia still could not be established in a recent study.^[19]^

## 6 Diagnosis

Due to the unclear manifestation of ASMAE and non-specific laboratory studies, this condition is often misdiagnosed causing serious morbidity and high mortality. As physicians, it is very significant for us to make a general judgment quickly with an accurate direction so that the ASMAE patients can complete the necessary accessory examinations to be diagnosed and treated timely, which is pivotal for patients’ survival and prognosis. It has been shown in Ibrahim's^[20]^ study that survival rate is about 27.1% when the diagnosis is delayed, but it increases to approximately 89.4% when the accurate diagnosis has been made within 24 hours after the onset of symptoms. With regards to the middle aged and elderly people with the pathogenesis of atrial fibrillation, atherosclerosis, coronary artery disease, rheumatic heart disease, infective endocarditis and more, they present with sudden onset severe abdominal pain accompanied by nausea and vomiting, ASMAE should be estimated and measures must be taken promptly.

It is important to remember that when intestinal ischemia is clinically suspected, diagnostic imaging studies should be performed. Mesenteric angiography is the gold standard in the past for the diagnosis of ASMAE, which was recommended by The American Gastroenterological Association.^[3]^ However, the increasingly extensive introduction and use of CTA with high quality has promoted the early diagnosis of ASMAE with a sensitivity of 93% and specificity of 96%.^[21]^ Nowadays, CTA is gradually going to supplant diagnostic angiography for three reasons. On 1 hand, CTA combining vascular and bowel assessment, such as pneumatosis, portal vein gas, focal edematous bowel wall, mesenteric edema, other solid organ infarction, results in a high diagnostic accuracy.^[22]^ Frank et al^[23]^ reported a sensitivity and specificity of CTA with the concomitant evaluation of the bowel for diagnosing AMI of 89.4% and 99.5%. On the other hand, CTA is much more universal so that patients have 24-hour access to high-resolution CTA. Last but not least, CTA allows early diagnosis and differentiation between occlusive and non-occlusive causes invasively, which is useful to direct therapeutic approach.

Aortic angiography can identify the location and extent of embolism, which has been traditionally the most reliable method to assess the presence of ASMAE. Although angiography still plays a pivotal role in diagnosing ASMAE, it is an invasive examination and its therapeutic role was strengthened with an ever-expanding list of endovascular treatments or adjuncts. Angiography can provide complementary or stand-alone treatment after diagnosis based on specific condition, including thrombolysis, injection of vasodilators and angioplasty with or without stenting.^[14]^ Angiography is now superseded by CTA.^[13]^ However, if CT scanning is inconclusive and there is strong clinical suspicion of AMI, angiography ought to be performed urgently to verify the diagnosis. What is more, the confirmatory diagnostic arteriogram can be finished in the operating room with the trained vascular surgeons and improved intraoperative fluoroscopy. In this way, precious time can be saved in treating this kind of challenging patients.

## 7 Treatment

In ASMAE, principal goals of management can be summarized with the 4"Rs": resuscitation, rapid diagnosis, early revascularization, and reassessment of bowel.^[24]^ Open surgery is the traditional treatment option, including embolectomy, endarterectomy, and bypass grafting.^[4]^ However, patients with ASMAE are always elderly with multiple comorbidities and bad nutritional status, open surgery is not the best choice. As a result, endovascular treatment is a more appealing option with minimal invasion since it has been used and developed for several decades.^[25]^ For the last 10 years, many reports have described successfully reperfusion of thromboembolic SMA occlusion via several endovascular strategies such as percutaneous aspiration embolectomy, thrombolysis, balloon thrombectomy, percutaneous transluminal angioplasty, primary SMA stenting and a combination of these therapies, sometimes followed by explorative laparotomy for resection of the infarcted bowel segment.^[25–27]^ It has also been reported that long-term survival after endovascular treatment was better than after open surgery.^[28]^

If the patient at an early stage, in which the course of disease is less than 12 hours, without any signs of peritonism or bowel necrosis and a suspected or proven embolus on CTA, endovascular thrombectomy with or without thrombolysis can be attempted. The endovascular technique is usually performed by a standard femoral or puncture. The catheter is advanced to the occluded superior mesenteric ischemia, guided by the CTA. A brachial approach is useful for cannulating the SMA, especially when occluded because of its acute take-off angle from the aorta. Angiography is able to confirm the site of occlusion, and the catheter is advanced as close as possible to the occlusion. Subsequent continuous administration of thrombolytic agents after angiography with appropriate dose adjustments according to clinical response for at least 24 hours is warranted. Sometimes, however, thrombolysis does not work for some old thrombus from atria. At the same time, caution should be taken as significant hypotension may occur if the catheter invades the aorta. The patient requires careful observation after the endovascular treatment. If signs of peritonism or clinical deterioration occurs during this period, laparotomy is indicated.^[29]^ However, a review of Swedish vascular registry recognized endovascular therapy as a better mortality rates of 1-month and 1-year rather than open surgery for AMI, although limited by direct surgery for exacerbated patients or patients under unsuccessful endovascular therapy, who would be transformed into the surgery.^[28]^

However, laparotomy is still the most preferred treatment for ASMAE patients, especially for those with signs of peritonism or bowel necrosis, whose purpose is to restore blood supply, reduce the resection area and determine the vitality of the bowel. At laparotomy, apparent necrotic areas of bowel can be resected, but potentially viable bowel is supposed to be left for approximately 30 minutes once perfusion has been established or reassessed at a later planned laparotomy. The key to the operation lies in the range of bowel and mesenteric excision. During the operation, surgeons ought to reduce the intestinal resection length and try to save the life of the possible animate bowel to avoid the occurrence of short bowel syndrome while insufficient resection area may lead to the second open surgery. Surgeons can estimate the intestinal viability by clinical judgments.

1. A normal and florid intestinal wall without serous hemorrhage.
2. Pulsatile artery.
3. The intestinal peristalsis stimulation by mechanical factors and heat is possible.

The accuracy of this method as reported by Bulkley is up to 89%.^[30]^

Recent knowledge has generated a multidisciplinary surgical management of ASMAE and a hybrid operating room with capability for both endovascular and open surgery, which may be helpful for saving precious time because the confirmatory diagnostic arteriogram can be finished in the operating room with the trained vascular surgeons and improved intraoperative fluoroscopy.^[25]^

## 8 Discussion

ASMAE is a surgical emergency with high mortality. The severe and acute-onset abdominal pain with little finding in physical examination, which cannot be relieved by antispasmodic agent, accompanied with gastrointestinal emptying symptoms, such as vomiting and diarrhea, should be considered as ASMAE. Patients with the history of atrial fibrillation, heart valve disease and atherosclerosis should be arranged for further examination. Laboratory findings are insensitive and unspecific. Abdominal plain radiograph and Duplex ultrasonography perhaps have positive findings, but the negative results of the above tests cannot rule out the ASMAE. Angiography and CTA, however, can provide a clear diagnosis sensitively and specifically. Endovascular approaches have been increasingly reported in multiple case series describing success with initial endovascular therapy followed by an open surgical intervention in some cases. Acuity of presentation, presence of bowel infarction, and risk factors may influence a planned treatment approach. The key to successful treatment of AMI involves early clinical recognition based on a detailed history and physical examination along with assessment of contributory risk factors, rapid acquisition of appropriate diagnostics to confirm a diagnosis, and early intervention to correct the underlying abnormality. In a word, physicians should strengthen the awareness of this disease to reduce the rate of misdiagnosis and save the precious time and lives of patients.

## Author contributions

**Methodology:** Haoyang Cao.

**Software:** Haoyang Cao.

**Writing - original draft:** Guanyi Liao.

**Writing - review & editing:** Guanyi Liao, Qing Gao, Siyang Chen, Wuwan Wang.
